# Recent Trends in Biosensors for Environmental Quality Monitoring

**DOI:** 10.3390/s22041513

**Published:** 2022-02-15

**Authors:** Simona Gavrilaș, Claudiu Ștefan Ursachi, Simona Perța-Crișan, Florentina-Daniela Munteanu

**Affiliations:** Faculty of Food Engineering, Tourism and Environmental Protection, “Aurel Vlaicu” University of Arad, Tourism and Environmental Protection, 2-4 E. Drăgoi Str., 310330 Arad, Romania; simona.gavrilas@uav.ro (S.G.); claudiu.ursachi@uav.ro (C.Ș.U.); simona.perta-crisan@uav.ro (S.P.-C.)

**Keywords:** environmental quality monitoring, emerging contaminants, detection, biosensing, mimetic biosensors

## Abstract

The monitoring of environmental pollution requires fast, reliable, cost-effective and small devices. This need explains the recent trends in the development of biosensing devices for pollutant detection. The present review aims to summarize the newest trends regarding the use of biosensors to detect environmental contaminants. Enzyme, whole cell, antibody, aptamer, and DNA-based biosensors and biomimetic sensors are discussed. We summarize their applicability to the detection of various pollutants and mention their constructive characteristics. Several detection principles are used in biosensor design: amperometry, conductometry, luminescence, etc. They differ in terms of rapidity, sensitivity, profitability, and design. Each one is characterized by specific selectivity and detection limits depending on the sensitive element. Mimetic biosensors are slowly gaining attention from researchers and users due to their advantages compared with classical ones. Further studies are necessary for the development of robust biosensing devices that can successfully be used for the detection of pollutants from complex matrices without prior sample preparation.

## 1. Introduction

The modern world faces a major problem today—environmental pollution, which is caused by the release and accumulation of various harmful substances due to current industries’ extreme development, rapid urbanization, and population growth. Pollutants are very diverse, ranging from chemical to physical, biological, and radiological compounds, and are widely spread in the air, soil, and waters, affecting all living systems, especially human health and life [1]. The safety and security of the environment is a major concern worldwide; therefore, prudent monitoring and management of it constitute two of the global and European priorities [2]. Researchers are interested in finding durable solutions to environmental monitoring, as the control of toxic substances is a fundamental condition for pollution remediation. Usually, the classical chromatographic [3,4,5] and spectroscopic [6,7,8,9] methods are used to detect contaminants, which are generally characterized by high sensibility and selectiveness. However, these methods are laborious, need several sample preparation steps, use toxic chemicals, and are time-consuming; and the equipment needs well-qualified operators.

The necessity of using some rapid, selective, sensitive, accurate, and real-time devices for detecting and screening pollutants led to the development of advanced biosensing devices. These must combine the analytical techniques with biotechnology in careful and reliable ways, at a low cost [10,11,12]. A special use of biosensors is in the evaluation of ecological risks. Biosensors are in such cases essential in complementing the specific chemical analyses [13,14]. For the construction of the biosensors should be considered the complexity of the environmental samples, as their use for technological applications is highly demanded [15,16,17].

Environmental pollutants can be monitored using specific biosensors. The detection principle must be based on a suitable physical/chemical transducer integrated with a compatible biological or biomimetic element that reversibly binds the analyte. The detector identifies and converts the resulting reactions into qualitative and quantitative sensing signals for the targeted pollutants from the sample [11,16].

The pollutants released from industrial, agricultural, and other intense human activities [11] are organic and inorganic. Biosensors’ usage is essential for monitoring actual conditions of soil, water, and air samples to detect pollutants such as pesticides, potentially toxic elements, pathogens, toxins, and endocrine-disrupting chemical compounds [2]. The major and long-lasting environmentally relevant toxicants can be separated into four categories: *organochlorine pesticides* (aldrin, chlordane, DDT (dichlorodiphenyltrichloroethane), dieldrin, endrin, heptachlor, mirex, and toxaphene); *fungicides* (i.e., hexachlorobenzene); *industrial chemicals* (PCBs—polychlorinated biphenyls and their by-products), and *heavy metals*. The possibility of their quantification by using specific biosensors constitutes a significant advantage in controlling them [11]. Even though biosensors have proved their abilities to measure air pollutants in various sample types, their efficiency is often poor [10].

The capacity of these small devices to offer reliable analytical results productively and profitably should be highlighted [18]. Another characteristic that needs to be underlined is the possibility offered by to perform ongoing in-field monitoring of various pollutants [19].

**Figure 1:**
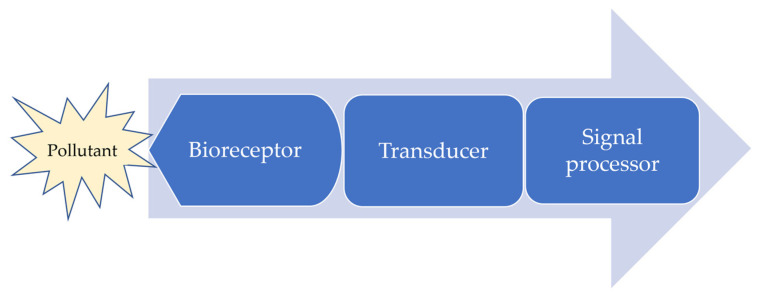
Operation of a biosensor.

Biosensors are analytical devices that each incorporate a biological sensing element to detect a targeted analyte from complex samples [20]. Biosensors convert a biological signal into a detectable electrical, optical, or thermal signal. They provide high sensitivity even with miniscule analyte concentrations [1,21,22]. A schematic diagram of the typical components of a biosensor is presented in Figure 1.

A biodetection device consists of some distinct components: a bioreceptor, a transducer, a system for signal processing, and a display [16,21]. The entire unit produces a measurable detection signal relating the analyte’s concentration in the target [23]. The biochemical receptor is used to recognize biological or chemical elements from the analyzed sample, being intimately associated with the transducing element, which converts the biochemical outcome into quantized electrical, optical, or thermal signal [21,22,24]. The biorecognition element might be a biological material, such as enzymes or a multienzyme system, microbes, recombinant microorganisms, functional nucleic acids, antibodies, antigens, aptamers, or an animal or plant tissue [21,24]. New alternatives use biomimetic materials (biomimetic catalysts, molecularly imprinted polymers, combinatorial ligands, etc.) [25]. Even if the biosensor is a complete, independent unit, the term specifically refers to the component that provides precise, complex bioanalytical measurements in simple formats and in real-time [10,20,24]. Biosensors must allow reuse and not be affected by pH and temperature [26].

**Figure 2:**
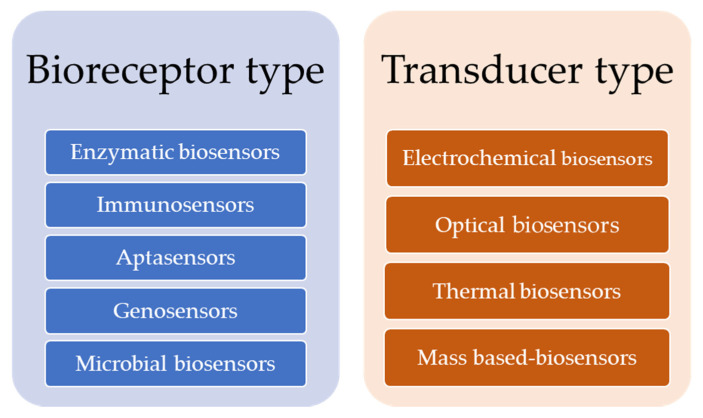
Classification of biosensors.

Biosensors are classified by the most important components involved in the detection process: the bioreceptor and the transducer. Regarding the bioreceptor type, biosensors can be grouped as follows: the biocatalytic group (enzymatic biosensors), the bioaffinity group (immunosensors, aptasensors, genosensors), and the microbial group (microbial biosensors) [2,26]. Based on the transducer’s physicochemical features and its working principle, biosensors are categorized as: electrochemical (potentiometric, amperometric, impedimetric, conductometric biosensors), optical (fiber-optic, surface plasmon resonance, Raman spectroscopy-based, and FTIR-based biosensors), and mass-based (magnetoelectric and piezoelectric biosensors) (Figure 2) [16].

Biosensors present some advantages in analytical chemistry. They expedite the processes of the traditional laboratory and analytical monitoring procedures—that is, taking various analytes from diverse samples. They are small and simple devices with high sensitivity and bioselectivity for targeted analytes, precision, rapidity, and continuity in monitoring. Several factors for users must also be considered when designing them, such as easy manipulation and operation, safety functioning, suitability for in situ detection (no complex sample preparation), real-time detection, cost efficiency, and eco-friendliness [27,28].

Biosensors have seen rapid and varied development in the past few decades [10] due to their ability to identify a wide range of analytes, such as pollutants, bacteria, fungi, drugs, and food additives [16]. Such attributes demonstrate their great applicability in various fields—pharmaceutics, medicine, industry, environmental monitoring, agriculture, food, forensic chemistry, security and defense, robotics, etc. [24,27]. The main uses of a biosensor depend on the specific tasks of the application area. Their utility in the food industry was demonstrated in quality and safety control, by discerning natural and artificial components, monitoring fermentation processes, etc. Their applicability in industry is mainly in control processes. In drug discovery and clinical and medical sciences, their use is recommended for rapidly detecting chemicals or viruses that cause various diseases, including cancer [20,26].

**Figure 3:**
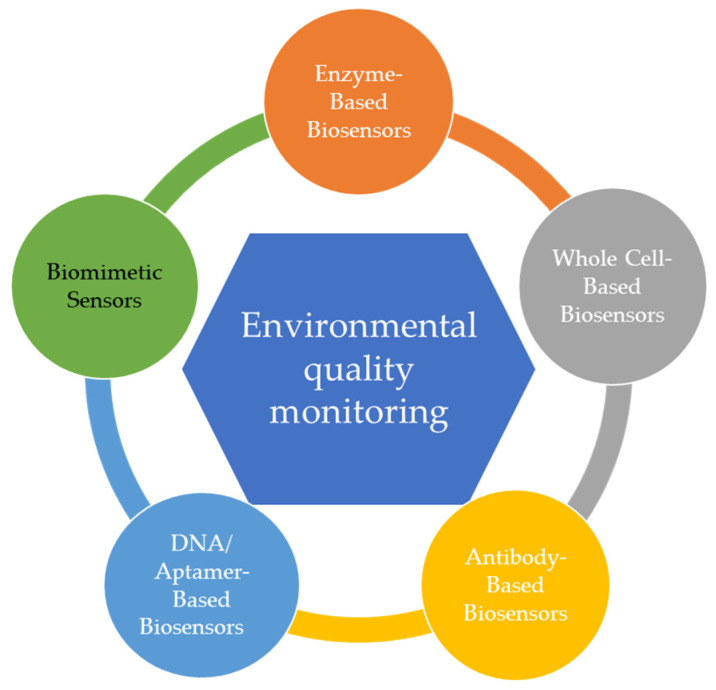
Biosensors used for the environmental quality monitoring.

Currently, there is increasing interest in developing highly accurate and efficient systems for identifying and screening environmental pollutants (Figure 3) [29].

Compared to other types of biosensors, e.g., biomedical ones, biosensors for environmental monitoring have a nonaged phase due to the complexity of the analysis, such as the complex ecological matrix, which interferes with pollutant recognition.

A biosensor’s characteristics are directly related to its biorecognition element and its transducer’s properties. Therefore, the materials used for the construction of the biosensor play an important role. Recently, laminated composites have become of great interest to various industries and applications [30,31,32,33,34,35,36,37,38,39,40,41,42]. The development of new composite materials is grabbing researchers’ attention, as these materials are characterized by high surface-to-volume ratios, high catalytic activity, good electrical conductivity, and good magnetic properties [43,44,45,46,47]. Yang et al. [47] extensively presented the synthesis of carbon nanotubes (CNT) (arc discharge, laser ablation, chemical vapor deposition (CVD), etc.) and the possibilities for their functionalization.

Nanocomposites represent a promising technology that enhances the sensitivity and flexibility of analyses of environmental complex samples. Nanostructures such as tubes, wires, rods, and particles modify biosensors’ characteristics toward achieving this goal. However, as Nigam et al. [10] noticed, there is still a real need for innovations in biosensors for environmental purposes, to assure high output of analysis for continuous, automated, and real-time results. Still, accuracy must also be considered the primary priority.

## 2. Sensors Used for Environmental Monitoring Overview

### 2.1. Enzyme-Based Biosensors

Enzymes are macromolecules with a complex 3D structure consisting of proteins that act as biological catalysts. An enzyme-based biosensor uses a specific enzyme as a biological sensing element, combined with a transducer that converts the signal generated by the enzymatic reaction into a measurable response proportional to the analyte concentration [48]. The enzymatic reaction signal can be generated in different forms: thermal release, proton concentration changes, oxygen emission or uptake, light emission or absorption, etc. The transducer (optical, electrochemical, thermal, piezoelectric) transforms this signal into potential, current, temperature exchange, light absorption, etc.—all of these being measurable by different means [49].

*Enzymatic biosensors have* earned massive interest in the last few years due to their multiple advantages, such as the high specificity and selectivity of enzymatic reactions, their wide range of detectable analytes, flexibility in detection, and the high purity of the available enzymes [50].

Naresh et al. [51] present in their paper the operating principles of enzymatic biosensors. There are two possible categories of mechanism of action: metabolization of the target analyte by the enzyme; or the activation, inhibition, or alteration of the enzyme by the analyte.

**Figure 4:**
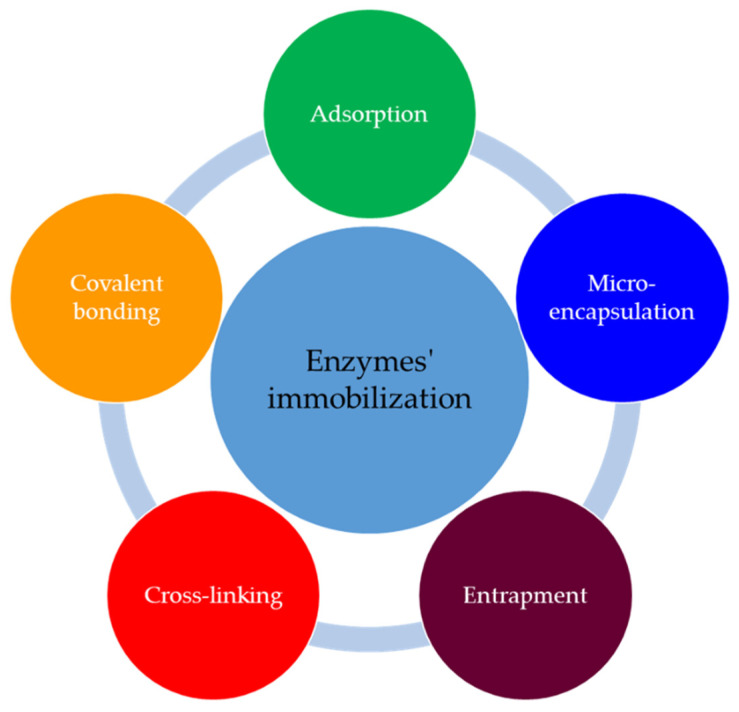
Methods for immobilization of the enzymes.

The essential requirements of an enzymatic biosensor are the immobilization the enzymes to the transducer’s surface and maintenance of their activity after immobilization [48]. The immobilized enzymes are more stable than the mobile versions and can be repetitively and continuously used [52]. The main methods for enzyme immobilization are presented in Figure 4, and in Table 1 are the characteristics of these.

Enzyme-based biosensors are widely used in food, medical, agricultural, and environmental fields. As shown in Table 2, the development of enzymatic biosensors for environment monitoring represents a subject of considerable interest.

### 2.2. Whole Cell-Based Biosensors (Microbial)

Whole-cell-based biosensors use natural or genetically engineered microorganisms (bacteria, fungi, algae, protozoa, or viruses) that can interact with a broad array of analytes and produce a signal detectable and quantifiable by a specific transducer [65]. Several transducers have been integrated with microorganisms, being built on different principles: electrical (amperometric, conductometric, potentiometric), colorimetric, and optical (colorimetric, luminescent, fluorescent) [66,67,68]. Microbial biosensors operate under a range of working conditions and are more sensitive to environmental signals than conventional ones [15]. They present various advantages: low limits of detection, high selectivity, and high sensitivity. Based on these features, whole-cell bioreceptors are applicable in many fields [51].

Microbial sensors can be considered a developed form of enzyme-based biosensors, as their mechanisms of detection are mostly identical. Both of them require the application of an immobilization technique to fix the biological material onto transducers or support matrices. As in the enzymes case, microorganisms can be immobilized by physical (adsorption and entrapment) and chemical methods (covalent binding and cross-linking). Finally, the chosen immobilization method must ensure mechanical resistance, cell viability, safe handling, and long-term storage [69].

Besides the advantages presented over the conventional methods, namely, high sensitivity, simultaneous detection of several compounds, high potential for on-site examinations, and cost-effectiveness, microbial biosensors are also associated with some drawbacks. Their long response times, the cells’ sensitivity to environmental variables (temperature, pH, etc.), and the difficulty of maintaining cell viability for an extended period are some of their limitations [15,65,70].

Numerous recent articles reported on the use of microbial biosensors to detect environmental pollutants, such as pesticides, heavy metals (As, Cu, Hg, Pb, or Cd), phenols, and other toxic compounds, using terrestrial and aquatic biota [15,19,71,72]. Other microbial biosensors were proposed and developed in the last few years as well, with remarkable applicability to environmental monitoring. Table 3 summarizes the results of several such investigations reported in the literature.

### 2.3. Antibody-Based Biosensors

Antibodies or immunoglobulins are a large class of glycoproteins produced by specialized cells as part of the immune system to detect harmful substances (antigens), such as microorganisms and chemicals. The antibodies can recognize and bind antigens, leading to stable antibody–antigen complexes [82,83,84]. Depending on how they are harvested, antibodies can be monoclonal or polyclonal. Monoclonal antibodies are laboratory-produced by hybridoma selection, whereas polyclonal antibodies are complex mixtures of antibodies isolated after animal immunization [85].

Antibody-based biosensors, also called immunosensors, are compact devices that detect and quantify, using a transducer, the specific interaction between immunoglobulins and antigens. Depending on the transducing mechanism, immunosensors are classified as electrochemical (amperometric, potentiometric, and impedimetric), colorimetric, optical, and microgravimetric. They can also be classified as labelled or nonlabelled sensors [17,86,87,88]. The labelling consists of attaching a sensitively detectable marker to the targeted analyte or the bioreceptor. During the analysis, the tag’s activity is measured. These tags may can be various sorts of compounds, including enzymes, fluorescent dyes, electroactive compounds, and nanoparticles [89]. Nonlabelled immunosensors are designed so that the antigen–antibody complex can be directly determined by estimating the physical changes produced by its development [51].

Immunosensors possess the advantages of better selectivity and sensitivity than classical analytical methods. At the same time, the evolution of immunoreactions on the detector’s surface can be observed in real-time [83,90]. However, the limitations in using antibody-based biosensors must also be considered, such as pH and temperature sensitivity, considerable time consumption, and the need for developing specialized reagents for each compound [91].

Several applications of the antibody-based biosensors within environmental monitoring are summarized in Table 4.

### 2.4. DNA/Aptamer-Based Biosensors

#### 2.4.1. Aptamer-Based Biosensors

Aptamers or “chemical antibodies” [99] are artificial, single-stranded oligonucleotide (DNA (deoxyribonucleic acid) or RNA (ribonucleic acid) sequences (15–80 base pairs in length) that can bind to specific target molecules [100]. The range of aptamer targets is extensive, from small molecules (peptides, proteins, carbohydrates, metal ions) to cells, viruses, and bacteria [101,102,103].

Aptamers can be selected in vitro through a process called SELEX (systematic evolution of ligands by exponential enrichment) [104,105,106]. The SELEX procedure starts with preparing an extensive library of oligonucleotides with different sequences, with which the target molecules are incubated for some time. After incubation, unbounded molecules are separated, and the target-bound oligonucleotides are eluted by heating or washing. The bound aptamer molecules are amplified by the polymerase chain reaction (PCR) to create the input for the following selection rounds. The entire process uses 5–15 cycles of selection and amplification [107,108,109].

In comparison with antibodies, aptamers have some specific advantages, such as higher stability in various environmental conditions (temperature, pH), lower cost, the ability to regenerate, and the possibility of being chemically synthesized or modified in accordance with target molecules [89,102,108].

In the last few years, several biosensors (colorimetric, fluorescent, electrochemical, and SERS—surface enhanced Raman spectroscopy) have been designed to detect environmental pollutants, using aptamers as the bioreceptors. Furthermore, the synthesis of new nanomaterials showed their significant potential for the development of innovative aptasensors. The latter are sustained by their strong biocompatibility with aptamers [102,106].

Table 5 summarizes recent studies on aptasensors developed for the detection of pollutants.

#### 2.4.2. DNA-Based Biosensors

DNA-based biosensors use nucleic acids (single-stranded DNA, ss-DNA) as recognition elements. Their working principle is based on two mechanisms: (i) the hybridization process between the target DNA and its complementary strand immobilized on a sensing area through the spontaneous hydrogen bonding between adenine–thymine and cytosine–guanine pairs [49,124]; (ii) the alteration of the ss-DNA structure by the target analyte’s molecules [125]. These mechanisms induce various physicochemical changes that lead to the generation of a specific signal that can be converted into a measurable response by an appropriate transducer, usually optical or electrochemical [126].

A significant stage in the design of DNA-based biosensors is the immobilization procedure of the nucleic acid fragments on the electrode surface. Regardless of the method used (adsorption, covalent bonding, or avidin–biotin interaction), the immobilization must preserve the activity of these fragments—that is, ensure their stability and accessibility to the target molecules [127].

Due to their multiple advantages, such as specificity, sensitivity, biocompatibility, and cost-effectivity, DNA-based biosensors are used in several fields, including disease prognosis, clinical diagnosis, food control, and environmental screening [126,128].

Several studies have illustrated the ability of DNA-based biosensors to detect traces of heavy metals in the environment [125,128,129,130]. In this case, the working principle is based on the affinity of some heavy metal ions toward forming stable duplex structures together with certain DNA bases. Mercury ion (Hg^2+^) selectively binds thymine (T) bases and creates a thermal stable T-Hg^2+^–T duplex [131]. Similarly, silver ions (Ag^+^) selectively interact with two cytosine (C) bases and form C–Ag^+^–C base pairs, which stabilize the DNA duplex [49,125]. Therefore, in the presence of some metal ions, thymine-rich or cytosine-rich single-stranded DNA can form stable structures by which metals can be detected with adequate transducers [125].

Some of the recent DNA-based biosensors’ applications are presented in Table 6.

## 3. Biomimetic Sensors

Although the terminology may seem new, the basis of biomimetics was laid years ago. Its principle is finding solutions that mimic a natural system’s mechanisms, especially regarding the structure of an organism or its specific interactions with the environment. The created products can be performant and adequately adapted to real environments [142].

Biomimetic sensors were first constructed while considering the basic principles of the related enzymatic biosensors. The intention was to maintain high sensibility, selectivity, sensitivity, and easy operation, while simultaneously decreasing some of the disadvantages. The limitations that need to be overcome mainly relate to each enzyme’s specific features, such as inactivation issues, or high costs because of the purification and standardization processes. In such contexts, the research was conducted toward finding sustainable solutions for creating imitative systems. Some of the developed models are based on metal complexes, molecularly imprinted polymers, nanozymes, synzymes, and nanochannels [143].

**Figure 5:**
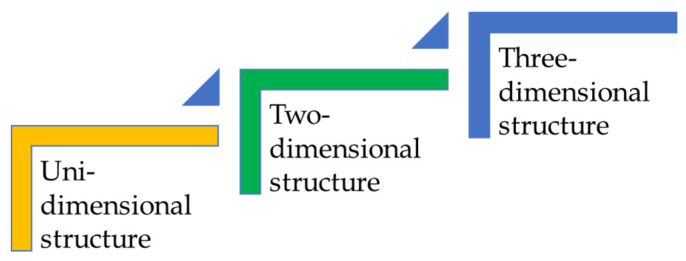
Structures used for the construction of biomimetic sensors.

In the last few years, the domain of biomimetic sensors has registered significant progress. Initially, biomimetic sensors were constructed using uni- or bi-dimensional structures (Figure 5). Then tridimensional assemblies were widely used, and the results indicated improved performances, sometimes exceeding the natural models’ performances [143]. Finding the proper ligand for the targeted analyte is the first step in designing precise tools. The peptide selection used in the recognition systems is important for the sensor’s affinity [144]. Computer modelling [145] and simulation are two stages that improve the performances of these devices.

The domain of biomimetic sensors used for environmental pollutants detection is currently developing. Research has opened multiple promising directions for the construction of such sensors: modified nanoparticles [146,147,148], metal chalcogenides nanocrystals built on various microorganisms [149], valorization of classical imprinted electrodes [150], and nanozymes for phenol removal [151].

Some examples of sensors created based on mimetic principles with applications in environmental monitoring are summarized in Table 7.

## 4. Future Perspectives

Another approach of biosensors regards the possibility of simultaneous detection of multiple pollutants. Several investigations have been successfully conducted to that end. Raymundo-Pereira et al. [164] evidenced the possibility of using carbon screen-printed electrodes for parallel identification of estradiol, paracetamol, and hydroquinone in tap water. Their findings could have an important application in wastewater analysis. Good prospects for use in water quality analysis were also provided by a luminescent sensor derived from a stable europium(III) metal–organic framework. It was tested for antibiotic identification [165]. The interest in using biosensors for water contaminant detection was also fostered by Martins et al. [166]. They identified sulfamethoxazole and trimethoprim from water samples.

The first steps toward making a biosensor with two detection mechanisms were made by Belaidi et al. [167]. Their electrochemical and optical detection biosensor, based on different algae responses, showed promising perspectives for simultaneous pesticide identification in water samples. These findings also provoked the design of a mimetic biosensor capable of detecting multiple pollutants.

The biosensors constructed for environmental quality monitoring will continue to be improved by using novel nanocomposites and nanomaterials, and new functionalization methods, but the necessity for in situ and real-time monitoring of pollutants will lead to the development of new sensing systems and even their coupling with aircraft systems [168].

With the current need for cheap, sensitive, fast, and reliable devices for environmental monitoring, the main challenge remains the gap between the results of academic research and the implementation of these biosensors as marketable products.

## 5. Conclusions

This review aimed to show that the need for fast, reliable, and stable devices for the detection of environmental pollutants can be satisfied by biosensors. However, these should answer the demands of sensitivity and selectivity when used in complex and unpredictable environmental samples with changeable compositions.

Independent of the sensing element or transducer, when developing biosensors for environmental pollutants detection, it is important to consider the possibility of continuous use, which would require fast renewal of the biological activity during the detection cycles; portability; cost; and last but not least, the possibility of automatization and integration into professional devices. In most investigations, the performance of the biosensor is assessed based on standardized laboratory samples.

The biological sensing elements—enzymes, aptamers, DNA, antibodies, and microorganisms—might face challenges in terms of stability, possible interference, and optimal working conditions, but these still have the advantage of being open to improvements in terms of specificity and selectivity.

As a result of scientific research in recent years, biomimetic sensors are characterized by better kinetic performances than enzyme-based biosensors. Still, specificity and selectivity remain their main shortcomings.

## Figures and Tables

**Table 1:** Methods of enzyme immobilization for biosensors [52,53].

Immobilization of Enzymes	Method’s Characteristics
Adsorption	Simple, inexpensive, less destructive to enzymatic activity, no additional reagent necessary
Microencapsulation	Preservation of structural and acting integrities of enzymes, due to their protection against environmental conditions
Entrapment	High stability conferred to the enzymes
Cross-linking	Improved efficiency and stability of enzymes by strong and stable bindings
Covalent bondings	More stability for enzymes and enzymes-support complexes, meanwhile stronger bindings than in adsorption case

**Table 2:** Examples of enzyme-based biosensors used for environmental monitoring.

Analyte	Enzyme(s)	Immobilization Method	Transducer	Target	LOD	Linearity	Reference
Hg^2+^, Cu^2+^, Cd^2+^	Urease	Entrapment in sol-gel matrix	Optical	River water	10 nM, 50 μM, 500 μM	-	[54]
Chromium	GOx	Cross-linking with GA and covering with aniline membrane	Amperometric	Soil	0.49 µg L^−1^	0.49–95.73 mgL^−^^1^ 95.73–8.05 mgL^−1^	[55]
Paraoxon	AChE	Dropping on the multiwall carbon nanotubes	Amperometric	Water	0.5 nmol L^−1^	6.9 nM	[56]
Paraoxon-ethyl, diisopropyl fluorophosphates	AChE	Cross-linking with BSA in a saturated glutaraldehyde vapor	Conductometric	Soil	1 × 10^−8^, 5 × 10^−11^	-	[57]
Atrazine	Tyrosinase	Cross-linking with PVA-SbQ	Amperometric	Spiked drinking water s	0.3 ppm	0.5–20 ppm	[58]
Atrazine	Tyrosinase	Entrapping in poly(L-DOPA)	Amperometric	Water	10 ppb	50 ppb–3.0 ppm	[59]
Organophosphorus neurotoxin	AChE	Cross-linking with GA	Piezoelectric	Water	50 mg/m^3^	0–50 mg/m^3^	[60]
Captan	Glutathione-S-transferase	Entrapment in gel sodium alginate	Optical	Water	2 ppm	-	[61]
Anatoxin-a	AChE	Entrapment in PVA-SbQ	Amperometric	Water	1 µg L^−1^	0–2.0 ppm	[62]
Catechol	Tyrosinase	Chitosan-gold nanoparticles	Amperometric	Environmental monitoring	27 × 10^−6^ mM	0.046–50 μM	[63]
Methyl salicylate	Alcohol oxidase and peroxidase	Molecular tetherings in carbon nanotube matrix	Amperometric	Environmental monitoring	0.00098 mM	-	[64]

Abbreviations: LOD—limit of detection; Gox—glucose oxidase; GA—glutaraldehyde; AchE—acetylcholinesterase; BSA—bovine serum albumin; PVA-SbQ—polyvinyl alcohol bearing styrylpyridinium groups; L-DOPA-l-3,4-dihydroxyphenylalanine.

**Table 3:** Examples of microbial biosensors used for environmental monitoring.

Analyte	Microorganism	Immobilization Method	Transducer	Target	LOD	Reference
As^3+^	Genetically engineered *S. oneidensis*	Biofilm formation	Electrochemical	Environmental monitoring	40 μM	[73]
Cu^2+^, Cd^2+^, Ni^2+,^ Pb^2+^	*Saccharomyces cerevisiae* S288C	Physical adsorption on BND-chitosan hydrogell polymer on GCE	Amperometric	Wastewater	-	[74]
As^3+^, Cd^2+^, Pb^2+^, Zn^2+^	*E. coli*	Microbial culture in microfluidic device	Fluorescent	Water	-	[75]
Pb^2+^	*E. Coli* DH5α	Microbial culture in a microfluidic device	Fluorescent	Environmental monitoring		[76]
Cd^2+^, Cu^2+^, Zn^2+^	*Bacillus megaterium* VR1	Entrapment in sol-gel matrix	Fluorescent	Soil	1.42 × 10^−4^, 3.16 × 10^−4^, 2.42 × 10^−4^	[14]
Cu^2+^	*S. Cerevisiae*	Entrapment in alginate beads	Colorimetric	Water	1 µM	[77]
Paraoxon, parathion, methylparathion	Genetically engineered *Escherihia coli*	Biofilm on GCE modified with OMCs	Amperometric	Environmmental monitoring	9 nM, 10 nM, 15 nMz	[78]
Atrazine (herbicide)	*Anabaena variabilis*	Entrapment in alginate	Amperometric	Environmmental monitoring	0.07 µM	[79]
Diuron (herbicide)	*Chlamydomonas reinhardtii*	Ti/TiO_2_ ultramicroe-lectrodes in algal suspension	Chronoamperometric	Water	0.2 µM	[80]
Simazine (herbicide)	*Dictyosphaerium chlorelloides* Dc1M	Adsorption on porous silicone disks	Luminescent	Drinking water	40.8 µg L^−1^	[81]

LOD—limit of detection; BND—boron-doped nanocrystalline diamond; GCE—glassy carbon electrode; OMCs—ordered mesopore carbons.

**Table 4:** Examples of immunosensors used for environmental monitoring.

Analyte	Transducer	Electrode/Sensing Material	Target	LOD	Linearity	Reference
Chlorpyrifos	Impedimetric	Chip modified with gold nanoparticles	-	0.5 ng mL^−^^1^	0.5–500 ng/ml	[92]
TBBPA-DHEE and TBBPA-MHEE	Impedimetric	Silica nanoparticles	Aquatic environments	0.08 ng mL^−^^1^	0.21–111.31 ng/mL	[93]
Atrazine	Electrochemical	SWCNT	Seawater, riverine water	0.01 ng mL^−^^1^	-	[94]
Microcystin-LR	Impedimetric	Gold electrodes with MoS2 andgold nanorods	Water	5 ng L^−^^1^	0.01–20 gL^−^^1^	[95]
Okadaic acid Domoic acid	Optical (SPR)	Gold electrode with carboxymethylated surface	Seawater	0.36 ng mL^−^^1^ 1.66 ng mL^−^^1^	-	[96]
Okadaic acid	Impedimetric	Graphene	Seawater	0.05 ng mL^−1^	-	[97]
*Legionella* *pneumophila*	Optical (SPR)	Gold substrate	Water	103 CFU mL^−1^	-	[98]

Abbreviations: TBBPA-DHEE—tetrabromobisphenol A bis(2-hydroxyethyl) ether; TBBPA-MHEE—tetrabromobisphenol A mono(hydroxyethyl) ether; SWCNT—single-walled carbon nanotubes; SPR—surface plasmon resonance; microcystin—LR-microcystin-leucinearginine.

**Table 5:** Examples of aptamer-based biosensors used for environmental monitoring.

Analyte	Detection Method	Target	LOD	Linearity	Reference
Ag^+^	SERS based on Au@Ag core–shell nanoparticles	Tap water, river water	50 × 10^−^^12^ mg L^−1^	0.1–100 nM	[110]
As^3+^	Colorimetric with GNPs	Wastewater	0.0006 mg L^−1^	1–400 range/ppm	[111]
As^3+^	Colorimetric with AuNPs	Soil	1.97 ppm	-	[112]
Cd^2+^	Fluorescence with use of SYBR green I as signal reporter	Tap water, river water	3 × 10^−^^9^ mg L^−1^	1.12–224.82 μg L^−1^	[113]
Hg^2+^	SERS based on dual recycling	Water environment	0.11 fM	0.2–125 fM	[114]
Hg^2+^	SERS based on SiO_2_@Au core/shell nanoparticles	Lake water	10 × 10^−^^9^ mg L^−1^	-	[115]
Pb^2+^	Electrochemical (Impedance), G-rich aptamer/MWCNTs/GNPs	Water	4.3 × 10^–^^15^ M	5.0 × 10^−^^11^–1.0 × 10^−^^14^ M	[116]
Pb^2+^	Fluorescence based on gold nanoflowers	Tap water	0.285 nM	0.01–850 nM	[117]
Pb^2+^	Colorimetric with use of silver staining	Soil	5.0 × 10^−^^7^ mg L^−1^	-	[118]
Acetampirid	Chemiluminescence with use of AuNPs	Wastewater Soil	62 × 10^−^^12^ mg L^−1^ 1.0 × 10^−^^9^ mg L^−1^	-	[119]
Malathion	Colorimetric based on AuNPs and cationic polymer	Lake water	6 × 10^−^^14^ mg L^−1^	0.5–1000 pM	[120]
Omethoate	Fluorescence based on S-GQD	-	1 ppb	0–200 ppm	[121]
Organophosphorus pesticides	Fluorescence with poly(T) CuNPs	Lake water	0.22 nM	0–200 nM	[122]
Tetracycline	Photoelectrochemical based on CdTe-BiOBr heterojunction	Soil	9.25 pM	10–1500 pM	[123]

Abbreviations: GNPs—gold nanoparticules; G—guanine; SERS—surface-enhanced Raman scattering; CuNPs—copper nanoparticles; S-GQD—sulphur-doped graphene quantum dot, SYBR—N′,N′-dimethyl-N-[4-[(E)-(3-methyl-1,3-benzothiazol-2-ylidene)methyl]-1-phenylquinolin-1-ium-2-yl]-N-propylpropane-1,3-diamine; G-rich—guanine-rich; MWCNTs— carboxylic acid group functionalized multiwalled carbon nanotubes (MWNTs-COOH).

**Table 6:** Examples of DNA-based biosensors used for environmental monitoring.

Analyte	Transducer	Target	LOD	Linearity	Reference
Hg^2+^	Electrochemical	Tap water, river water	0.05 nM	0.1–200 nM	[132]
Pb^2+^	Fluorescent	Aqueous systems	5 nM	0–50 nM	[133]
Pb^2+^	Fluorescent	Lake water	0.6 nM	2–10 nM	[134]
Organophosphorus pesticides	Fluorescent	Lake water	0.018 µg L^−1^	2–10 μg/L	[134]
Cyanazine	Impedimetric	Water	0.8 nM	4.0 nM–70 μM	[135]
Pirazon	Impedimetric	Water	1 × 10^−^^10^ M	5 × 10^−9^–5 × 10^−5^ M	[136]
*Legionella pneumophila*	Optical (SPRi)	Water	104 CFU mL^−^^1^	-	[137]
*Vibrio cholerae*	Impedimetric	-	7.41 × 10^−^^30^ mol L^−1^	10^−^^8^–10^−^^14^ mol L^−1^	[138]
*Escherichia coli*	Amperometric	Soil	100 cells/g soil	-	[139]
*Bacillus thuringiensis*	Impedimetric	-	0.997 × 10^−^^12^ M	1 pM–1 μM	[140]
*Ostreopsis* cf. *ovata*	Colorimetric	Plankton, bentonite	9 pg/μL	-	[141]

Abbreviations: SPR—surface plasmon resonance imaging; CFU—colony-forming units.

**Table 7:** Examples of biomimetic sensors used for environmental monitoring.

Analyte	Mimetic Structure	Transducer	Target	Sensibility (LOD)	Linearity	Reference
	Heavy metals					
Cu^2+^, Cr^3+^, Fe^3+^, Pb^2+^, Fe^2+^, Cd^2+^, Cr^6+^, Co^2+^, Zn^2+^, Ag^+^, Al^3+^	Enzyme immobilization Metal phosphates-acetylcholinesterase nanoflowers	Colorimetric	Water	Cu^2+^—0.81 μM, Cr^3+^—0.75 μM Al^3+^—1.06 μM	2.5–500 μM.	[152]
Pb^2+^	Gold nanoparticles with glutathione linker	UV–vis spectroscopic	Water	47.6 nM (9.9 ppb)	2–14 mM	[153]
Hg^2+^	Cysteine-decorated ferromagnetic particle (Cys-Fe_3_O_4_)	Colorimetric	River water	5.9 pM.	0.02–90 nM	[154]
	Chemicals					
Methyl green	Magnetic molecularly imprinted polymer	Square-wave adsorptive anodic stripping voltammetry	River waterIndustrial wastewater	1.0 × 10^−8^ mol L^−1^	9.9 × 10^−^^8^–1.8 × 10^−^^6^ mol L^−^^1^	[145]
Acetylcholinesterase inhibitors	Microchannel 1-phenyl-1,2,3-butanetrione 2-oxime (PBO)-based microsensor	Potentiometric	Surface waters used for municipal drinking water supplies	LD50, LC50	2–1360 mg kg^−^^1^	[155]
Acetone gas	Zeolitic imidazolate framework-90 polyhedron crystals	quartz crystal microbalance	Air	Lower than 20 ppb	-	[156]
Nitrite ions	Oxo-bridged dinuclear manganese-phenanthroline complex immobilized into an ion-exchange Polymeric film deposited on glassy carbon electrode	Cyclic voltammetry	Environmental samples	6.50 × 10^−6^ mol L^−1^	2.49 × 10^−6^–9.90 × 10^−6^ mol L^−1^	[157]
Catechol	Metal-organic frameworks		Water	33 nmol L^−1^	-	[158]
Urea	Embedding urease and bovine hemoglobin in metal-organic frameworks through biomimetic mineralization	Colorimetric	Sewage	0.02 mM	0.08–20.00 mM	[159]
	Pesticides					
Diurone	Carbon paste electrode modified with the nickel(II) 1,4,8,11,15,18,22,25-octabutoxy-29*H*,31*H*-phthalocyanine complex	Cyclic voltammetry and amperometry	River water, soil	6.14 × 10^−6^ mol L^−1^,	9.9 ×10^−^^6^ and 1.5 × 10^−4^ mol L^−1^	[160]
Organophosphorus pesticides	Employing a functionalized polyacrylamide, polyhydroxamicalkanoate	Amperometric	Water supply	0.26 μmol L^−1^	-	[161]
Carbamate	Gold nanoclusters-anchored MnO_2_ (AuNCs-MnO_2_) nanocomposite	Fluorimetric/Colorimetric	Soil, water	0.125 µg L^−^^1^.	-	[162]
Paraoxon	Cu_3_(PO_4_)_2_·3H_2_O, AChE and ChO -based lab-on paper platform	Cyclic voltammetry and Colorimetric	Tap and river water	6 fg mL^−^^1^	-	[163]
	Toxins					
Bacterial toxins	Microcystins inserted into a polymeric matrix	Potentiometric	Water	below the guideline value establishedby WHO	7.24 × 10^−10^–1.28 × 10^−9^ M	[150]

Abbreviations: LOD—limit of detection; LD50—lethal dose (50%); LC50—lethal concentration (50%); WHO—World Health Organization; Cys—cysteine.

## Data Availability

Not applicable.

## References

[1] Xiong J., Sun Z., Yu J.H., Liu H., Wang X.D. (2022). Thermal self-regulatory smart biosensor based on horseradish peroxidase-immobilized phase-change microcapsules for enhancing detection of hazardous substances. Chem. Eng. J..

[2] Justino C.I.L., Duarte A.C., Rocha-Santos T.A.P. (2017). Recent Progress in Biosensors for Environmental Monitoring: A Review. Sensors.

[3] Deng F., Zhang D., Yang L., Li L., Lu Y., Wang J., Fan Y., Zhu Y., Li X., Zhang Y. (2021). Effects of antibiotics and heavy metals on denitrification in shallow eutrophic lakes. Chemosphere.

[4] Li L., He J., Gan Z., Yang P. (2021). Occurrence and fate of antibiotics and heavy metals in sewage treatment plants and risk assessment of reclaimed water in Chengdu, China. Chemosphere.

[5] Wu W., Qu S., Nel W., Ji J. (2022). Tracing and quantifying the sources of heavy metals in the upper and middle reaches of the Pearl River Basin: New insights from Sr-Nd-Pb multi-isotopic systems. Chemosphere.

[6] Brunnbauer L., Gonzalez J., Lohninger H., Bode J., Vogt C., Nelhiebel M., Larisegger S., Limbeck A. (2021). Strategies for trace metal quantification in polymer samples with an unknown matrix using Laser-Induced Breakdown Spectroscopy. Spectrochim. Acta Part B At. Spectrosc..

[7] Trapananti A., Eisenmann T., Giuli G., Mueller F., Moretti A., Passerini S., Bresser D. (2021). Isovalent vs. aliovalent transition metal doping of zinc oxide lithium-ion battery anodes—In-depth investigation by ex situ and operando X-ray absorption spectroscopy. Mater. Today Chem..

[8] Dhote S.S., Deshmukh L., Paliwal L. (2013). Miceller chromatographic method for the separation of heavy metal ions and spectrophotometric estimation of UO_2_^2+^ on bismuth silicate layer. Int. J. Chem. Anal. Sci..

[9] Murzyn C.M., Allen D.J., Baca A.N., Ching M.L., Marinis R.T. (2021). Tunable Infrared Laser Absorption Spectroscopy of Aluminum Monoxide A2Πi−X2Σ+. J. Quant. Spectrosc. Radiat. Transf..

[10] Nigam V.K., Shukla P. (2015). Enzyme Based Biosensors for Detection of Environmental Pollutants—A Review. J. Microbiol. Biotechnol..

[11] Khanam Z., Gupta S., Verma A. (2020). Endophytic fungi-based biosensors for environmental contaminants—A perspective. S. Afr. J. Bot..

[12] Kumar T., Naik S., Jujjavarappu S.E. (2021). A critical review on early-warning electrochemical system on microbial fuel cell-based biosensor for on-site water quality monitoring. Chemosphere.

[13] Ivask A., Green T., Polyak B., Mor A., Kahru A., Virta M., Marks R. (2007). Fibre-optic bacterial biosensors and their application for the analysis of bioavailable Hg and As in soils and sediments from Aznalcollar mining area in Spain. Biosens. Bioelectron..

[14] Rathnayake I.V.N., Megharaj M., Naidu R. (2021). Green fluorescent protein based whole cell bacterial biosensor for the detection of bioavailable heavy metals in soil environment. Environ. Technol. Innov..

[15] Bilal M., Iqbal H.M.N. (2019). Microbial-derived biosensors for monitoring environmental contaminants: Recent advances and future outlook. Process Saf. Environ. Prot..

[16] Kumar H., Kumari N., Sharma R. (2020). Nanocomposites (conducting polymer and nanoparticles) based electrochemical biosensor for the detection of environment pollutant: Its issues and challenges. Environ. Impact Assess. Rev..

[17] Tschmelak J., Proll G., Riedt J., Kaiser J., Kraemmer P., Barzaga L., Wilkinson J.S., Hua P., Hole J.P., Nudd R. (2005). Biosensors for unattended, cost-effective and continuous monitoring of environmental pollution: Automated Water Analyser Computer Supported System (AWACSS) and River Analyser (RIANA). J. Environ. Anal. Chem..

[18] Hashem A., Hossain M.A.M., Marlinda A.R., Mamun M.A., Simarani K., Johan M.R. (2021). Nanomaterials based electrochemical nucleic acid biosensors for environmental monitoring: A review. Appl. Surf. Sci. Adv..

[19] Chung T.H., Meshref M.N.A., Dhar B.R. (2021). A review and roadmap for developing microbial electrochemical cell-based biosensors for recalcitrant environmental contaminants, emphasis on aromatic compounds. Chem. Eng. J..

[20] Turner A.P.F. (2013). Biosensors: Sense and sensibility. Chem. Soc. Rev..

[21] Jain U., Saxena K., Hooda V., Balayan S., Singh A.P., Tikadar M., Chauhan N. (2022). Emerging vistas on pesticides detection based on electrochemical biosensors—An update. Food Chem..

[22] Chen C., Wang J.S. (2020). Optical biosensors: An exhaustive and comprehensive review. Analyst.

[23] Sethi R.S. (1991). Transducer aspects of biosensors. GEC J. Res..

[24] Mehrotra P. (2016). Biosensors and their applications—A review. J. Oral Biol. Craniofac Res..

[25] Abbasian F., Ghafar-Zadeh E., Magierowski S. (2018). Microbiological sensing technologies: A review. Bioengineering.

[26] Chen Y.Y., Liu J.C., Yang Z.C., Wilkinson J.S., Zhou X.H. (2019). Optical biosensors based on refractometric sensing schemes: A review. Biosens. Bioelectron..

[27] Badihi-Mossberg M., Buchner V., Rishpon J. (2007). Electrochemical Biosensors for pollutants in the environment. Electroanalysis.

[28] Asif S., Chaudhari A., Gireesh-Babu P., Chaudhuri P.R., Sen R. Immobilization of fluorescent whole cell biosensors for the improved detection of heavy metal pollutants present in aquatic environment. Proceedings of the International Conference on Advances in Bioprocess Engineering and Technology (ICABET).

[29] Huang H.P., Chen Y.A., Chen Z.Z., Chen J.L., Hu Y.M., Zhu J.J. (2021). Electrochemical sensor based on Ce-MOF/carbon nanotube composite for the simultaneous discrimination of hydroquinone and catechol. J. Hazard. Mater..

[30] Lubineau G., Rahaman A. (2012). A review of strategies for improving the degradation properties of laminated continuous-fiber/epoxy composites with carbon-based nanoreinforcements. Carbon.

[31] Martins P., Lanceros-Mendez S. (2013). Polymer-Based Magnetoelectric Materials. Adv. Funct. Mater..

[32] Nikbakt S., Kamarian S., Shakeri M. (2018). A review on optimization of composite structures Part I: Laminated composites. Compos. Struct..

[33] Porras A., Maranon A. (2012). Development and characterization of a laminate composite material from polylactic acid (PLA) and woven bamboo fabric. Compos. Part B-Eng..

[34] Sahay R., Kumar P.S., Sridhar R., Sundaramurthy J., Venugopal J., Mhaisalkar S.G., Ramakrishna S. (2012). Electrospun composite nanofibers and their multifaceted applications. J. Mater. Chem..

[35] Tornabene F., Viola E., Fantuzzi N. (2013). General higher-order equivalent single layer theory for free vibrations of doubly-curved laminated composite shells and panels. Compos. Struct..

[36] Treviso A., Van Genechten B., Mundo D., Tournour M. (2015). Damping in composite materials: Properties and models. Compos. Ptart B-Eng..

[37] Tang L., Dang J., He M.K., Li J.Y., Kong J., Tang Y.S., Gu J.W. (2019). Preparation and properties of cyanate-based wave-transparent laminated composites reinforced by dopamine/POSS functionalized Kevlar cloth. Compos. Sci. Technol..

[38] Tang L., He M.K., Na X.Y., Guan X.F., Zhang R.H., Zhang J.L., Gu J.W. (2019). Functionalized glass fibers cloth/spherical BN fillers/epoxy laminated composites with excellent thermal conductivities and electrical insulation properties. Compos. Commun..

[39] Tang Y.S., Dong W.C., Tang L., Zhang Y.K., Kong J., Gu J.W. (2018). Fabrication and investigations on the polydopamine/KH-560 functionalized PBO fibers/cyanate ester wave-transparent composites. Compos. Commun..

[40] Wang G.L., Yu D.M., Kelkar A.D., Zhang L.F. (2017). Electrospun nanofiber: Emerging reinforcing filler in polymer matrix composite materials. Prog. Polym. Sci..

[41] Wang J., Zhou Q., Shao S., Misra A. (2017). Strength and plasticity of nanolaminated materials. Mater. Res. Lett..

[42] Yahaya R., Sapuan S.M., Jawaid M., Leman Z., Zainudin E.S. (2015). Effect of layering sequence and chemical treatment on the mechanical properties of woven kenaf-aramid hybrid laminated composites. Mater. Des..

[43] Yoon J., Shin M., Lee T., Choi J.W. (2020). Highly sensitive biosensors based on biomolecules and functional nanomaterials depending on the types of nanomaterials: A perspective review. Materials.

[44] Kurbanoglu S., Ozkan S.A., Merkoçi A. (2017). Nanomaterials-based enzyme electrochemical biosensors operating through inhibition for biosensing applications. Biosens. Bioelectron..

[45] Cavalcante F.T.T., de Falcão I.R.A., da Souza J.E.S., Rocha T.G., de Sousa I.G., Cavalcante A.L.G., de Oliveira A.L.B., de Sousa M.C.M., dos Santos J.C.S. (2021). Designing of Nanomaterials-Based Enzymatic Biosensors: Synthesis, Properties, and Applications. Electrochem.

[46] Gaviria-Arroyave M.I., Cano J.B., Peñuela G.A. (2020). Nanomaterial-based fluorescent biosensors for monitoring environmental pollutants: A critical review. Talanta Open.

[47] Yang N., Chen X., Ren T., Zhang P., Yang D. (2015). Carbon nanotube based biosensors. Sens. Actuators B Chem..

[48] Rahimi P., Joseph Y. (2019). Enzyme-based biosensors for choline analysis: A review. TrAC Trends Anal. Chem..

[49] Asal M., Ozen O., Sahinler M., Polatoglu I. (2018). Recent Developments in Enzyme, DNA and Immuno-Based Biosensors. Sensors.

[50] Economou A., Karapetis S.K., Nikoleli G.-P., Nikolelis D.P., Bratakou S., Varzakas T.H., Toldrá F., Nollet L.M.L. (2017). Enzyme-Based Sensors. Advances in Food Diagnostics.

[51] Naresh V., Lee N. (2021). A Review on Biosensors and Recent Development of Nanostructured Materials-Enabled Biosensors. Sensors.

[52] Nguyen H.H., Lee S.H., Lee U.J., Fermin C.D., Kim M. (2019). Immobilized Enzymes in Biosensor Applications. Materials.

[53] Nguyen H.H., Kim M. (2017). An Overview of Techniques in Enzyme Immobilization. Appl. Sci. Converg. Technol..

[54] Amine A., Mohammadi H., Bourais I., Palleschi G. (2006). Enzyme inhibition-based biosensors for food safety and environmental monitoring. Biosens. Bioelectron..

[55] Zeng G.M., Tang L., Shen G.L., Huang G.H., Niu C.G. (2004). Determination of trace chromium(VI) by an inhibition-based enzyme biosensor incorporating an electropolymerized aniline membrane and ferrocene as electron transfer mediator. J. Environ. Anal. Chem..

[56] Joshi K.A., Tang J., Haddon R., Wang J., Chen W., Mulchandani A. (2005). A disposable biosensor for organophosphorus nerve agents based on carbon nanotubes modified thick film strip electrode. Electroanalysis.

[57] Dzyadevych S.V., Soldatkin A.P., Arkhypova V.N., El’skaya A.V., Chovelon J.M., Georgiou C.A., Martelet C., Jaffrezic-Renault N. (2005). Early-warning electrochemical biosensor system for environmental monitoring based on enzyme inhibition. Sens. Actuators B Chem..

[58] Tortolini C., Bollella P., Antiochia R., Favero G., Mazzei F. (2016). Inhibition-based biosensor for atrazine detection. Sens. Actuators B Chem..

[59] Guan Y., Liu L., Chen C., Kang X., Xie Q. (2016). Effective immobilization of tyrosinase via enzyme catalytic polymerization of l-DOPA for highly sensitive phenol and atrazine sensing. Talanta.

[60] Tang S., Ma W.Y., Xie G.Z., Su Y.J., Jiang Y.D. (2016). Acetylcholinesterase-reduced graphene oxide hybrid films for organophosphorus neurotoxin sensing via quartz crystal microbalance. Chem. Phys. Lett..

[61] Devic E., Li D.H., Dauta A., Henriksen P., Codd G.A., Marty J.L., Fournier D. (2002). Detection of anatoxin-a(s) in environmental samples of cyanobacteria by using a biosensor with engineered acetylcholinesterases. Appl. Environ. Microbiol..

[62] Choi J.-W., Kim Y.-K., Song S.-Y., Lee I.-h., Lee W.H. (2003). Optical biosensor consisting of glutathione-S-transferase for detection of captan. Biosens. Bioelectron..

[63] Polatoglu İ., Kızılkaya M., Eren Ü. (2016). Development of a gold nanoparticle based electrochemical biosensor for detection of phenolic compounds. J. Turk. Chem. Soc. Sect. B Chem. Eng..

[64] Fang Y., Umasankar Y., Ramasamy R.P. (2016). A novel bi-enzyme electrochemical biosensor for selective and sensitive determination of methyl salicylate. Biosens. Bioelectron..

[65] Chang H.J., Voyvodic P.L., Zuniga A., Bonnet J. (2017). Microbially derived biosensors for diagnosis, monitoring and epidemiology. Microb. Biotechnol..

[66] Lei Y., Chen W., Mulchandani A. (2006). Microbial biosensors. Anal. Chim. Acta.

[67] D’Souza S.F. (2001). Microbial biosensors. Biosens. Bioelectron..

[68] Chung T.H., Dhar B.R. (2021). Paper-based platforms for microbial electrochemical cell-based biosensors: A review. Biosens. Bioelectron..

[69] Moraskie M., Roshid M.H.O., O’Connor G., Dikici E., Zingg J.-M., Deo S., Daunert S. (2021). Microbial whole-cell biosensors: Current applications, challenges, and future perspectives. Biosens. Bioelectron..

[70] Lim J.W., Ha D., Lee J., Lee S.K., Kim T. (2015). Review of Micro/Nanotechnologies for Microbial Biosensors. Front. Bioeng. Biotechnol..

[71] Gupta N., Renugopalakrishnan V., Liepmann D., Paulmurugan R., Malhotra B.D. (2019). Cell-based biosensors: Recent trends, challenges and future perspectives. Biosens. Bioelectron..

[72] Do M.H., Ngo H.H., Guo W., Chang S.W., Nguyen D.D., Pandey A., Sharma P., Varjani S., Nguyen T.A.H., Hoang N.B. (2021). A dual chamber microbial fuel cell based biosensor for monitoring copper and arsenic in municipal wastewater. Sci. Total Environ..

[73] Webster D.P., TerAvest M.A., Doud D.F.R., Chakravorty A., Holmes E.C., Radens C.M., Sureka S., Gralnick J.A., Angenent L.T. (2014). An arsenic-specific biosensor with genetically engineered Shewanella oneidensis in a bioelectrochemical system. Biosens. Bioelectron..

[74] Gao G.Y., Fang D.Y., Yu Y., Wu L.Z., Wang Y., Zhi J.F. (2017). A double-mediator based whole cell electrochemical biosensor for acute biotoxicity assessment of wastewater. Talanta.

[75] Kim H.J., Lim J.W., Jeong H., Lee S.-J., Lee D.-W., Kim T., Lee S.J. (2016). Development of a highly specific and sensitive cadmium and lead microbial biosensor using synthetic CadC-T7 genetic circuitry. Biosens. Bioelectron..

[76] Bae J., Lim J.-W., Kim T. (2018). Reusable and storable whole-cell microbial biosensors with a microchemostat platform for in situ on-demand heavy metal detection. Sens. Actuators B Chem..

[77] Vopálenská I., Váchová L., Palková Z. (2015). New biosensor for detection of copper ions in water based on immobilized genetically modified yeast cells. Biosens. Bioelectron..

[78] Tang X., Zhang T., Liang B., Han D., Zeng L., Zheng C., Li T., Wei M., Liu A. (2014). Sensitive electrochemical microbial biosensor for p-nitrophenylorganophosphates based on electrode modified with cell surface-displayed organophosphorus hydrolase and ordered mesopore carbons. Biosens. Bioelectron..

[79] Tucci M., Grattieri M., Schievano A., Cristiani P., Minteer S.D. (2019). Microbial amperometric biosensor for online herbicide detection: Photocurrent inhibition of Anabaena variabilis. Electrochim. Acta.

[80] Tsopela A., Laborde A., Salvagnac L., Ventalon V., Bedel-Pereira E., Seguy I., Temple-Boyer P., Juneau P., Izquierdo R., Launay J. (2016). Development of a lab-on-chip electrochemical biosensor for water quality analysis based on microalgal photosynthesis. Biosens. Bioelectron..

[81] Haigh-Flórez D., de la Hera C., Costas E., Orellana G. (2014). Microalgae dual-head biosensors for selective detection of herbicides with fiber-optic luminescent O_2_ transduction. Biosens. Bioelectron..

[82] Sharma S., Byrne H., O’Kennedy R.J., Estrela P. (2016). Antibodies and Antibody-Derived Analytical Biosensors. Biosensor Technologies for Detection of Biomolecules.

[83] Felix F.S., Angnes L. (2018). Electrochemical immunosensors—A powerful tool for analytical applications. Biosens. Bioelectron..

[84] Omidfar K., Khorsand F., Azizi M.D. (2013). New analytical applications of gold nanoparticles as label in antibody based sensors. Biosens. Bioelectron..

[85] Cristea C., Florea A., Tertiș M., Săndulescu R. (2015). Immunosensors. Biosensors-Micro and Nanoscale Applications.

[86] Fang L., Liao X., Jia B., Shi L., Kang L., Zhou L., Kong W. (2020). Recent progress in immunosensors for pesticides. Biosens. Bioelectron..

[87] Farka Z., Juriik T., Kovaar D., Trnkova L., Sklaadal P. (2017). Nanoparticle-Based Immunochemical Biosensors and Assays: Recent Advances and Challenges. Chem. Rev..

[88] Shao Y.N., Zhou H., Wu Q.P., Xiong Y.H., Wang J., Ding Y. (2021). Recent advances in enzyme-enhanced immunosensors. Biotechnol. Adv..

[89] Rhouati A., Catanante G., Nunes G., Hayat A., Marty J.-L. (2016). Label-Free Aptasensors for the Detection of Mycotoxins. Sensors.

[90] Jia M.X., Liao X.F., Fang L., Jia B.Y., Liu M., Li D.H., Zhou L.D., Kong W.J. (2021). Recent advances on immunosensors for mycotoxins in foods and other commodities. TrAC—Trends Anal. Chem..

[91] Rogers K.R. (2006). Recent advances in biosensor techniques for environmental monitoring. Anal. Chim. Acta.

[92] Jia H.Y., Guo Y.M., Sun X., Wang X.Y. (2015). An Electrochemical Immunosensor Based on Microfluidic Chip for Detection of Chlorpyrifos. Int. J. Electrochem. Sci..

[93] Zhang Z., Dong S., Ge D., Zhu N., Wang K., Zhu G., Xu W., Xu H. (2018). An ultrasensitive competitive immunosensor using silica nanoparticles as an enzyme carrier for simultaneous impedimetric detection of tetrabromobisphenol A bis(2-hydroxyethyl) ether and tetrabromobisphenol A mono(hydroxyethyl) ether. Biosens. Bioelectron..

[94] Belkhamssa N., Justino C.I.L., Santos P.S.M., Cardoso S., Lopes I., Duarte A.C., Rocha-Santos T., Ksibi M. (2016). Label-free disposable immunosensor for detection of atrazine. Talanta.

[95] Zhang Y., Chen M., Li H., Yan F., Pang P., Wang H., Wu Z., Yang W. (2017). A molybdenum disulfide/gold nanorod composite-based electrochemical immunosensor for sensitive and quantitative detection of microcystin-LR in environmental samples. Sens. Actuators B Chem..

[96] McNamee S.E., Elliott C.T., Delahaut P., Campbell K. (2013). Multiplex biotoxin surface plasmon resonance method for marine biotoxins in algal and seawater samples. Environ. Sci. Pollut. Res..

[97] Antunes J., Justino C., da Costa J.P., Cardoso S., Duarte A.C., Rocha-Santos T. (2018). Graphene immunosensors for okadaic acid detection in seawater. Microchem. J..

[98] Enrico D.L., Manera M.G., Montagna G., Cimaglia F., Chiesa M., Poltronieri P., Santino A., Rella R. (2013). SPR based immunosensor for detection of Legionella pneumophila in water samples. Opt. Commun..

[99] Huang L., Wang X., Liu S., Gao Z.-X. (2021). Application of Aptamer-based Biosensor in Bisphenol A Detection. Chin. J. Anal. Chem..

[100] Wang T., Chen C.Y., Larcher L.M., Barrero R.A., Veedu R.N. (2019). Three decades of nucleic acid aptamer technologies: Lessons learned, progress and opportunities on aptamer development. Biotechnol. Adv..

[101] Monosik R., Stredanský M., Sturdik E. (2012). Biosensors-classification, characterization and new trends. Acta Chim. Slovaca.

[102] Wang K., Tao Z.-H., Xu L., Liu Y.-Q. (2014). Research and Development of Functionalized Aptamer based Biosensor. Chin. J. Anal. Chem..

[103] Muhammad M., Huang Q. (2021). A review of aptamer-based SERS biosensors: Design strategies and applications. Talanta.

[104] Nguyen V.T., Kwon Y.S., Gu M.B. (2017). Aptamer-based environmental biosensors for small molecule contaminants. Curr. Opin. Biotechnol..

[105] McConnell E.M., Nguyen J., Li Y.F. (2020). Aptamer-Based Biosensors for Environmental Monitoring. Front. Chem..

[106] Xie M.J., Zhao F.G., Zhang Y.P., Xiong Y., Han S.Y. (2022). Recent advances in aptamer-based optical and electrochemical biosensors for detection of pesticides and veterinary drugs. Food Control.

[107] Mishra G.K., Sharma V., Mishra R.K. (2018). Electrochemical Aptasensors for Food and Environmental Safeguarding: A Review. Biosensors.

[108] Komarova N., Kuznetsov A. (2019). Inside the Black Box: What Makes SELEX Better?. Molecules.

[109] Lyu C., Khan I.M., Wang Z.P. (2021). Capture-SELEX for aptamer selection: A short review. Talanta.

[110] Wu Y., Jiang T.T., Wu Z.Y., Yu R.Q. (2018). Internal standard-based SERS aptasensor for ultrasensitive quantitative detection of Ag+ ion. Talanta.

[111] Wu Y.G., Liu L., Zhan S.S., Wang F.Z., Zhou P. (2012). Ultrasensitive aptamer biosensor for arsenic(III) detection in aqueous solution based on surfactant-induced aggregation of gold nanoparticles. Analyst.

[112] Siddiqui M.F., Khan Z.A., Jeon H., Park S. (2020). SPE based soil processing and aptasensor integrated detection system for rapid on site screening of arsenic contamination in soil. Ecotoxicol. Environ. Saf..

[113] Zhou B., Yang X.Y., Wang Y.S., Yi J.C., Zeng Z., Zhang H., Chen Y.T., Hu X.J., Suo Q.L. (2019). Label-free fluorescent aptasensor of Cd^2+^ detection based on the conformational switching of aptamer probe and SYBR green I. Microchem. J..

[114] Tian C., Zhao L., Zhu J., Zhang S.S. (2021). Ultrasensitive detection of trace Hg^2+^ by SERS aptasensor based on dual recycling amplification in water environment. J. Hazard. Mater..

[115] Lu Y.L., Zhong J., Yao G.H., Huang Q. (2018). A label-free SERS approach to quantitative and selective detection of mercury (II) based on DNA aptamer-modified SiO_2_@Au core/shell nanoparticles. Sens. Actuators B Chem..

[116] Zhu Y., Zeng G.M., Zhang Y., Tang L., Chen J., Cheng M., Zhang L.H., He L., Guo Y., He X.X. (2014). Highly sensitive electrochemical sensor using a MWCNTs/GNPs-modified electrode for lead (II) detection based on Pb^2+^-induced G-rich DNA conformation. Analyst.

[117] Liu R., He B., Jin H., Suo Z. (2021). A fluorescent aptasensor for Pb^2+^ detection based on gold nanoflowers and RecJf exonuclease-induced signal amplification. Anal. Chim. Acta.

[118] Ma L.H., Wang H.B., Fang B.Y., Tan F., Cao Y.C., Zhao Y.D. (2018). Visual detection of trace lead ion based on aptamer and silver staining nano-metal composite. Colloids Surf. B.

[119] Qi Y.Y., Xiu F.R., Zheng M.F., Li B.X. (2016). A simple and rapid chemiluminescence aptasensor for acetamiprid in contaminated samples: Sensitivity, selectivity and mechanism. Biosens. Bioelectron..

[120] Bala R., Kumar M., Bansal K., Sharma R.K., Wangoo N. (2016). Ultrasensitive aptamer biosensor for malathion detection based on cationic polymer and gold nanoparticles. Biosens. Bioelectron..

[121] Nair R.V., Chandran P.R., Mohamed A.P., Pillai S. (2021). Sulphur-doped graphene quantum dot based fluorescent turn-on aptasensor for selective and ultrasensitive detection of omethoate. Anal. Chim. Acta.

[122] Zhao Y.W., Wang Y., Yang R.M., Zhang H., Zhao Y.F., Miao X.M., Lu L.H. (2021). A zero-background fluorescent aptasensor for ultrasensitive detection of pesticides based on magnetic three-dimensional DNA walker and poly(T)-templated copper nanoparticles. Sens. Actuators B Chem..

[123] Guo Z.J., Jiang K.T., Jiang H.H., Zhang H., Liu Q., You T.Y. (2022). Photoelectrochemical aptasensor for sensitive detection of tetracycline in soil based on CdTe-BiOBr heterojunction: Improved photoactivity enabled by Z-scheme electron transfer pathway. J. Hazard. Mater..

[124] Kokkinos C. (2019). Electrochemical DNA Biosensors Based on Labeling with Nanoparticles. Nanomaterials.

[125] Saidur M.R., Aziz A.R.A., Basirun W.J. (2017). Recent advances in DNA-based electrochemical biosensors for heavy metal ion detection: A review. Biosens. Bioelectron..

[126] Bacchu M.S., Ali M.R., Das S., Akter S., Sakamoto H., Suye S.I., Rahman M.M., Campbell K., Khan M.Z.H. (2021). A DNA functionalized advanced electrochemical biosensor for identification of the foodborne pathogen Salmonella enterica serovar Typhi in real samples. Anal. Chim. Acta.

[127] Hamed K.-K., Vahideh R., Ali E., Fatemeh S. (2020). DNA Biosensors Techniques and Their Applications in Food Safety, Environmental Protection and Biomedical Research: A mini-review. J. Cell Dev. Biol..

[128] Wang Q., Wang J., Huang Y., Du Y.C., Zhang Y., Cui Y.X., Kong D.M. (2022). Development of the DNA-based biosensors for high performance in detection of molecular biomarkers: More rapid, sensitive, and universal. Biosens. Bioelectron..

[129] Sun C.T., Ou X.W., Cheng Y., Zhai T.Y., Liu B.F., Lou X.D., Xia F. (2019). Coordination-induced structural changes of DNA-based optical and electrochemical sensors for metal ions detection. Dalton Trans..

[130] He Z.Y., Yin H.L., Chang C.C., Wang G.Q., Liang X.G. (2020). Interfacing DNA with Gold Nanoparticles for Heavy Metal Detection. Biosensors.

[131] Ono A., Togashi H. (2004). Highly selective oligonucleotide-based sensor for mercury(II) in aqueous solutions. Angew. Chem. Int. Ed..

[132] Zhang Y.Y., Zhang C., Ma R., Du X., Dong W.H., Chen Y., Chen Q. (2017). An ultra-sensitive Au nanoparticles functionalized DNA biosensor for electrochemical sensing of mercury ions. Mater. Sci. Eng. C Mater. Biol. Appl..

[133] Ravikumar A., Panneerselvam P., Radhakrishnan K., Morad N., Anuradha C.D., Sivanesan S. (2017). DNAzyme Based Amplified Biosensor on Ultrasensitive Fluorescence Detection of Pb (II) Ions from Aqueous System. J. Fluoresc..

[134] Radhakrishnan K., Kumar P.S. (2022). Target-receptive structural switching of ssDNA as selective and sensitive biosensor for subsequent detection of toxic Pb^2+^ and organophosphorus pesticide. Chemosphere.

[135] Karimi-Maleh H., Karimi F., Fu L., Sanati A.L., Alizadeh M., Karaman C., Orooji Y. (2022). Cyanazine herbicide monitoring as a hazardous substance by a DNA nanostructure biosensor. J. Hazard. Mater..

[136] Peyman H., Roshanfekr H., Ansari S. (2020). DNA-based electrochemical biosensor using chitosan-carbon nanotubes composite film for biodetection of Pirazon. Eurasian Chem. Commun..

[137] Foudeh A.M., Trigui H., Mendis N., Faucher S.P., Veres T., Tabrizian M. (2015). Rapid and specific SPRi detection of L-pneumophila in complex environmental water samples. Anal. Bioanal. Chem..

[138] Ali M.R., Bacchu M.S., Setu M.A.A., Akter S., Hasan M.N., Chowdhury F.T., Rahman M.M., Ahommed M.S., Khan M.Z.H. (2021). Development of an advanced DNA biosensor for pathogenic Vibrio cholerae detection in real sample. Biosens. Bioelectron..

[139] Rochelet M., Vienney F., Solanas S., Membrilla A., Hartmann A. (2013). An electrochemical DNA biosensor for the detection of CTX-M extended-spectrum β-lactamase-producing Escherichia coli in soil samples. J. Microbiol. Methods.

[140] Raju V.M., Bhavana V., Gayathri G.K., Suryan S., Reddy R., Reddy N., Ravikumar C.R., Santosh M.S. (2020). A novel disposable electrochemical DNA biosensor for the rapid detection of Bacillus thuringiensis. Microchem. J..

[141] Toldra A., Alcaraz C., Diogene J., O’Sullivan C.K., Campas M. (2019). Detection of Ostreopsis cf. ovata in environmental samples using an electrochemical DNA-based biosensor. Sci. Total Environ..

[142] Arriaza-Echanes C., Campo-Giraldo J.L., Quezada C.P., Espinoza-González R., Rivas-Álvarez P., Pacheco M., Bravo D., Pérez-Donoso J.M. (2021). Biomimetic synthesis of CuInS2 nanoparticles: Characterization, cytotoxicity, and application in quantum dots sensitized solar cells. Arab. J. Chem..

[143] Romanholo P.V.V., Razzino C.A., Raymundo-Pereira P.A., Prado T.M., Machado S.A.S., Sgobbi L.F. (2021). Biomimetic electrochemical sensors: New horizons and challenges in biosensing applications. Biosens. Bioelectron..

[144] Lowe C.R. (1999). Chemoselective biosensors. Curr. Opin. Chem. Biol..

[145] Khan S., Wong A., Zanoni M.V.B., Sotomayor M.D.P.T. (2019). Electrochemical sensors based on biomimetic magnetic molecularly imprinted polymer for selective quantification of methyl green in environmental samples. Mater. Sci. Eng. C.

[146] Yang S., Liu M., Deng F., Mao L., Yuan Y., Huang H., Chen J., Liu L., Zhang X., Wei Y. (2020). Biomimetic modification of silica nanoparticles for highly sensitive and ultrafast detection of DNA and Ag+ ions. Appl. Surf. Sci..

[147] Nasir M., Rauf S., Muhammad N., Nawaz M.H., Chaudhry A.A., Malik M.H., Shahid S.A., Hayat A. (2017). Biomimetic nitrogen doped titania nanoparticles as a colorimetric platform for hydrogen peroxide detection. J. Colloid Interface Sci..

[148] Cao W., Ju P., Wang Z., Zhang Y., Zhai X., Jiang F., Sun C. (2020). Colorimetric detection of H_2_O_2_ based on the enhanced peroxidase mimetic activity of nanoparticles decorated Ce_2_(WO_4_)_3_ nanosheets. Spectrochim. Acta A Mol. Biomol. Spectrosc..

[149] Vena M.P., Jobbagy M., Bilmes S.A. (2016). Microorganism mediated biosynthesis of metal chalcogenides; a powerful tool to transform toxic effluents into functional nanomaterials. Sci. Total Environ..

[150] Queirós R.B., Guedes A., Marques P., Noronha J., Sales M.G.F., Chemical A.B. (2013). Recycling old screen-printed electrodes with newly designed plastic antibodies on the wall of carbon nanotubes as sensory element for in situ detection of bacterial toxins in water. Sensors.

[151] Zhang S.Q., Lin F.F., Yuan Q.P., Liu J.W., Li Y., Liang H. (2020). Robust magnetic laccase-mimicking nanozyme for oxidizing o-phenylenediamine and removing phenolic pollutants. J. Environ. Sci..

[152] Zhai C., Miao L., Zhang Y., Zhang L., Li H., Zhang S. (2022). An enzyme response-regulated colorimetric assay for pattern recognition sensing application using biomimetic inorganic-protein hybrid nanoflowers. Chem. Eng. J..

[153] Chu W., Zhang Y., Li D., Barrow C.J., Wang H., Yang W. (2015). A biomimetic sensor for the detection of lead in water. Biosens. Bioelectron..

[154] Niu X.H., He Y.F., Li X., Zhao H.L., Pan J.M., Qiu F.X., Lan M.B. (2019). A peroxidase-mimicking nanosensor with Hg^2+^-triggered enzymatic activity of cysteine-decorated ferromagnetic particles for ultrasensitive Hg^2+^ detection in environmental and biological fluids. Sens. Actuators B Chem..

[155] Wujcik E.K., Londoño N.J., Duirk S.E., Monty C.N., Masel R.I. (2013). An acetylcholinesterase-inspired biomimetic toxicity sensor. Chemosphere.

[156] Zhang D., Fan Y., Li G., Du W., Li R., Liu Y., Cheng Z., Xu J. (2020). Biomimetic synthesis of zeolitic imidazolate frameworks and their application in high performance acetone gas sensors. Sens. Actuators B Chem..

[157] Machini W.B.S., Teixeira M.F.S. (2015). Application of oxo-manganese complex immobilized on ion-exchange polymeric film as biomimetic sensor for nitrite ions. Sens. Actuators B Chem..

[158] Cao Q., Xiao Y., Liu N., Huang R., Ye C., Huang C., Liu H., Han G., Wu L. (2021). Synthesis of Yolk/Shell heterostructures MOF@MOF as biomimetic sensing platform for catechol detection. Sens. Actuators B Chem..

[159] Cheng Y., Chen T., Fu D., Liu J. (2022). A molecularly imprinted nanoreactor based on biomimetic mineralization of bi-enzymes for specific detection of urea and its analogues. Sens. Actuators B Chem..

[160] Wong A., de Vasconcelos Lanza M.R., Sotomayor M.D.P.T. (2013). Sensor for diuron quantitation based on the P450 biomimetic catalyst nickel(II) 1,4,8,11,15,18,22,25-octabutoxy-29H,31H-phthalocyanine. J. Electroanal. Chem..

[161] Sgobbi L.F., Machado S.A.S. (2018). Functionalized polyacrylamide as an acetylcholinesterase-inspired biomimetic device for electrochemical sensing of organophosphorus pesticides. Biosens. Bioelectron..

[162] Yan X., Kong D., Jin R., Zhao X., Li H., Liu F., Lin Y., Lu G.J.S., Chemical A.B. (2019). Fluorometric and colorimetric analysis of carbamate pesticide via enzyme-triggered decomposition of Gold nanoclusters-anchored MnO_2_ nanocomposite. Sensors Actuators B Chem..

[163] Jin R., Kong D.S., Zhao X., Li H.X., Yan X., Liu F.M., Sun P., Du D., Lin Y.H., Lu G.Y. (2019). Tandem catalysis driven by enzymes directed hybrid nanoflowers for on-site ultrasensitive detection of organophosphorus pesticide. Biosens. Bioelectron..

[164] Raymundo-Pereira P.A., Gomes N.O., Machado S.A.S., Oliveira O.N. (2019). Simultaneous, ultrasensitive detection of hydroquinone, paracetamol and estradiol for quality control of tap water with a simple electrochemical method. J. Electroanal. Chem..

[165] Li L., Zou J.-Y., Zhang L., You S.-Y., Xie X., Chen G.-H. (2022). Sensitive detection of the antibiotic pollutants by a solvent-stable luminescent sensor based on a europium(III) metal-organic framework. J. Solid State Chem..

[166] Martins T.S., Bott-Neto J.L., Oliveira O.N., Machado S.A.S. (2021). Paper-based electrochemical sensors with reduced graphene nanoribbons for simultaneous detection of sulfamethoxazole and trimethoprim in water samples. J. Electroanal. Chem..

[167] Sekli Belaïdi F., Farouil L., Salvagnac L., Temple-Boyer P., Séguy I., Heully J.L., Alary F., Bedel-Pereira E., Launay J. (2019). Towards integrated multi-sensor platform using dual electrochemical and optical detection for on-site pollutant detection in water. Biosens. Bioelectron..

[168] Lu Y., Macias D., Dean Z.S., Kreger N.R., Wong P.K. (2015). A UAV-mounted whole cell biosensor system for environmental monitoring applications. IEEE Trans. Nanobiosci..

